# The high costs of getting ethical and site-specific approvals for multi-centre research

**DOI:** 10.1186/s41073-016-0023-6

**Published:** 2016-12-07

**Authors:** Adrian G. Barnett, Megan J. Campbell, Carla Shield, Alison Farrington, Lisa Hall, Katie Page, Anne Gardner, Brett G. Mitchell, Nicholas Graves

**Affiliations:** 1 Institute of Health and Biomedical Innovation & School of Public Health and Social Work, Queensland University of Technology, 60 Musk Avenue, Brisbane, Queensland 4059 Australia; 2 School of Nursing, Midwifery, and Paramedicine, Australian Catholic University, Canberra, Australia; 3 Faculty of Arts, Nursing and Theology, Avondale College for Higher Education, Wahroonga, New South Wales Australia

**Keywords:** Research ethics, Ethical review, Multi-centre study, Australia

## Abstract

**Background:**

Multi-centre studies generally cost more than single-centre studies because of larger sample sizes and the need for multiple ethical approvals. Multi-centre studies include clinical trials, clinical quality registries, observational studies and implementation studies. We examined the costs of two large Australian multi-centre studies in obtaining ethical and site-specific approvals.

**Methods:**

We collected data on staff time spent on approvals and expressed the overall cost as a percent of the total budget.

**Results:**

The total costs of gaining approval were 38 % of the budget for a study of 50 centres (mean cost AUD $6960 per site) and 2 % for a study of 11 centres (mean cost AUD $2300 per site). Seventy-five and 90 % of time was spent on repeated tasks, respectively, and many time-consuming tasks, such as reformatting documents, did nothing to improve the study design or participant safety.

**Conclusions:**

Improvements have been made to the ethical approval application system, but more gains could be made without increasing risks of harm to research participants. We propose that ethical review bodies and individual sites publish statistics on how long they take to process approvals which could then be nationally benchmarked.

## Background

An increasingly popular study design is a multi-centre study, in which a study protocol is simultaneously run over multiple sites. Many multi-centre studies are clinical trials, but they can also be quasi-experimental designs such as a pre-post design investigating the impact of a change in practice, or implementation studies examining how a proven implementation works at multiple sites, or clinical quality registries that collate routine outcome data from multiple sites.

Searching *PubMed*, there were 614 papers with “multi-centre trial/study” in the title in 2005 (including US spelling and no hyphen) and 1248 papers in 2015, which is a doubling in published multi-centre studies in 10 years. In the international clinical trials registry *ClinicalTrials.gov*, there were 1235 multi-centre studies registered to begin recruitment in 2005, and 2418 in 2015 (a 96 % growth). This rapid growth in multi-centre research was acknowledged by the chair of the Australian Health Ethics Committees [1].

Two common reasons for needing a multi-centre study are (1) to increase generalisability by including sites in multiple locations and/or of multiple types, for example, private and public hospitals in every county, state or province, and (2) where the site is the unit of randomisation and hence multiple sites are needed to provide adequate statistical power, for example, an intervention to reduce falls that applies to the whole hospital.

To ensure research on humans is ethically conducted, researchers must submit their detailed research plans to an ethical review body. In Australia, the submission requires multiple forms, including, but not limited to:

- A main application form such as the National Ethics Application Form (or NEAF) with questions on risk to participants and data security
- The consent form for participants
- The forms used to engage participants (e.g. information sheets, recruitment materials)
- All surveys or detailed protocols for clinical data or specimen collection

The ethical review body decides if the research meets the requirements of the Australian National Statement on Ethical Conduct in Human Research and is ethically acceptable [2]. In Australia, in addition to ethical review, there is a governance process carried out by each institution that permits research to be conducted under their auspices. In public health organisations, this is referred to as site-specific assessment and involves a separate set of forms. The application forms vary by site, but generally include details on recruitment, vulnerable groups, data security, resource use, budgets and legal implications. Both the ethics and site-specific forms usually require multiple signatures from the researchers, senior staff at their institution, and executive leaders at each participating site.

Additional ethics applications (to an ethical review body) are required for each site in a multi-centre study that is not covered by the initial ethics application. This is then followed by further site-specific approvals for each study site. Therefore, the more sites that are recruited the greater the ethics preparation time needed before the study can start. Since 2007, New South Wales allows one ethical review body to give multi-centre ethics approval [3]. In 2013, a “national” mutual acceptance scheme for single ethical review of multi-centre clinical trials was adopted by four states (New South Wales, Queensland, South Australia and Victoria). In 2015, the scheme was expanded to include all human research, although there are a number of important exceptions including projects requiring access to state-wide data collections, and research at universities and private hospitals. In Australia and in many other countries, all multi-centre studies must still complete multiple site-specific approval applications.

Our research team has recently managed two large national multi-centre studies and wanted to share our experience with the research community. We are concerned that the current process of getting ethical and site-specific approval is overly costly in terms of staff and time, and does not lead to any benefit, such as reducing risk for participants, as much of the time is spent on repeat applications [4]. Previous studies have examined the time taken for ethical review, and a recent international scoping review found 54 studies that examined the time taken and over 20 studies that examined costs [5]. We present two case studies and estimate the percent of the research budget spent on ethical and site-specific approvals.

## Methods

We examined the ethical and site-specific procedures for two multi-centre studies funded by the Australian National Health and Medical Research Council (NHMRC). The characteristics of the two studies are in Table 1. The NHHE was an observational study of hand hygiene practices in 50 hospitals. REACH was a stepped-wedged randomised trial of a cleaning intervention to reduce hospital-acquired infection in 11 hospitals. As neither study was a clinical trial, a mutual acceptance process was not available. Complete details on both studies are available in their protocol papers [6, 7].

**Table 1:** Characteristics of the two studies and the ethical requirements

Short title	National Hand Hygiene Evaluation (NHHE)	REACH Trial
Full title	Evaluating hand hygiene interventions and their ability to reduce healthcare associated infection	REACH: Researching Effective Approaches to Cleaning in Hospitals
Total funding (AUD)	$908,849	$1,146,817
Study years	2010–2013	2014–2017
Study design	Observational	Stepped-wedge randomised trial
Number of hospitals	50	11
States/territories	8	7
Number of ethical review bodies	24	11
Ethical risk category	High risk in Queensland (9 sites) and low risk in others	Low risk in all sites

We aimed to estimate the total time spent on getting ethical and site-specific approval both in terms of staff time and calendar time. We did not consider the time spent by the chief investigators writing the protocol or original grant application because our focus was on the approval process and the impact on the study budget.

For the REACH Trial, we prospectively collected data by asking staff members to note their time spent on ethical and site-specific approvals using an Excel spreadsheet with times split by each ethical review body and site. Ethics tasks included completing the application form, changing documents (logos, ethics complaints statements, footers), uploading documents to web sites, and email and phone conversations with ethical review body staff on application requirements (for documents, signatures). Site-specific tasks included completing the separate application form, changing documents (footers), uploading documents to web sites, and email and phone conversations with RGO staff on application requirements (for documents, signatures).

For the REACH Trial, we also recorded the dates of the start and end of the application process for each site. The start date was the date we started preparing the application. The end date was when approval was granted, including the time needed for any additional requests (e.g. confidentially agreements).

For the National Hand Hygiene Evaluation, we used a retrospective estimate of cost based on the staff members involved and the percentages of their time spent on ethics and site-specific applications.

For both studies, the cost estimates are relatively simple. We estimated the total time and salary costs and expressed the costs as a percent of the overall study budget. We estimated the proportion of time spent on repeat applications, that is, any time not on the original ethical or site-specific application.

## Results

The staff members and the percentages of their times on approvals are in Table 2. Four staff members worked on the National Hand Hygiene Evaluation. The overall cost of getting approvals was an estimated AUD $348,000 or 38 % of the total study budget. One notable application was for an ethical review body that required 30 hard copies of the application paperwork, including the 200-page NEAF application—over 7000 pages in total—and this ethical review body was aware that the application had already been approved by another ethical review body. An estimated 25 % of time was spent on the original ethics application, with 75 % on repeated applications. The planned timeline for ethical and site-specific approval underestimated the actual time by 17 months.

**Table 2:** Staff time and salary spent on ethical and site-specific approval for the two studies

Study	Staff member	Study year (PT for part-time)	Time (h)	Salary cost AUD $ (’000s)
National Hand Hygiene Evaluation	N1	Year 1	1322	81
		Year 2	353	23
		Year 3	353	25
	N2	Year 1 (PT 8 months)	705	31
		Year 2	1410	66
	N3	Year 3	1763	81
	N4	Year 3	881	41
Total			6786	348
REACH Trial	R1	Year 1	113	6
		Year 2	38	2
	R2	Year 1 (PT 2 months)	102	4
		Year 2 (PT)	348	13
Total			601	25

Two staff members were used for the REACH Trial (Table 2). The overall costs were estimated as AUD $25,000 or 2 % of the total study budget. Just 10 % of time was spent on the original ethics application, with the remaining 90 % on repeat ethics forms and site-specific approvals.

**Fig. 1:**
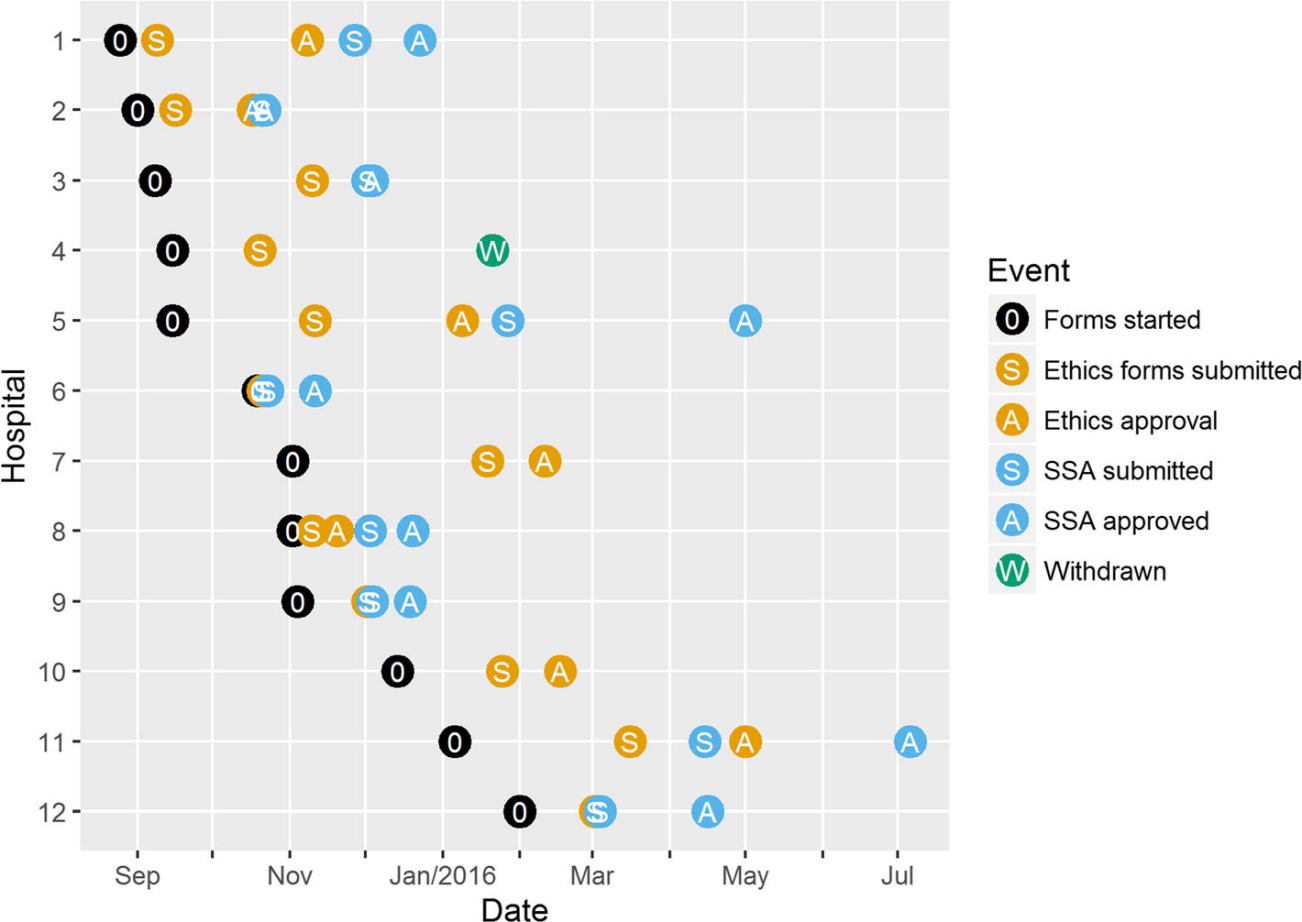
Application times by calendar date for the REACH Trial for the 12 hospitals (August 2015 to July 2016). One hospital was withdrawn. Two hospitals (7 and 10) did not need site-specific approval, one because the details were covered in the ethics forms and the other simply did not require a SSA. One ethics approval covered two sites

The calendar times of the applications for the REACH Trial are in Fig. 1. The overall process took 313 days, starting on 27 August 2015 and ending on 4 July 2016. The shortest approval time was 23 days for Hospital 6 and the longest was 229 days for Hospital 11, which is almost 10 times longer than the shortest. We printed over 2000 pages of application hard copy forms and 220 pages were electronic. Our planned time line for ethical and site-specific approval underestimated the actual time by 6 months.

The mean cost per site was AUD $2300 for REACH and AUD $6960 for the National Hand Hygiene study.

## Discussion

Our results show the potentially high costs of running multi-centre studies that require multiple approvals. Similarly, high costs in Australia were found by a multi-centre registry [8] and a multi-centre low risk observational study [9].

The costs were particularly high for the National Hand Hygiene Evaluation which covered 50 hospitals across 8 states and territories and needed 38 % of the budget, whereas the REACH Trial with 11 hospitals across 7 states and territories needed 2 % of the budget. Both studies were mostly low risk; high risk multi-centre studies could expect higher costs and longer delays [7]. Most of our staff members had experience with ethics applications, and studies using less experienced staff would likely experience longer delays. For NHHE, the work on ethical processes continued into the third year and for REACH into the second year, highlighting that this process is unlikely to be completed in the first year. Our original timelines for both studies underestimated the amount of time needed by at least 6 months. The delays meant that both projects had to apply to the funding agency for a timeline extension.

A number of processes created difficulties for both studies. One was the frequent change in regulatory staff which slowed the approval process when e-mails to staff who had left were lost and new staff needed time to become acquainted with our study. There was also variability in the speed with which regulatory staff responded to both general enquiries and submitted applications, possibly due to their experience and workload. Lack of clear, consistent or available guidelines for application requirements also hindered the process. Much of our time was spent on repetitive and low value tasks, such as reformatting documents for each new ethical review body’s requirements, uploading these documents to web sites, and obtaining signatures from researchers who had already signed multiple previous applications. These tasks incur costs and return no benefits in terms of improved study design or participant safety. Interestingly, the site-specific approval often took longer than the ethics application (Fig. 1), which is disappointing given that this process was designed to streamline the approval process.

In the REACH Trial, which could only commence once all sites had all required governance approvals, the variation in ethical review body processes and timings, and the unpredictable nature of the approval process, delayed trial commencement. The associated and protracted uncertainty of trial commencement was an issue for sites that had agreed early to participate and had already completed their approvals. In addition, staff costs continued throughout the extended approval process, with associated budget impact on trial implementation.

A key reason why REACH cost less than NHHE was because there were far fewer sites (11 compared with 50) and all REACH sites were classified as low risk whereas some NHHE sites were deemed to be high risk by some ethical review bodies. However, the REACH study was also cheaper per site. This could be because we employed a research assistant dedicated to leading the governance processes, and because of the lessons our research team learned in the earlier NHHE study. However, a recent study of using an ethics officer to speed applications found no impact on approval times [10]. There may also have been some streamlining of ethical processes over time, with some ethical review bodies and sites actively seeking to make the process easier for researchers.

It could be argued that the time and money was well spent as the public need to be protected from unethical research. However, for both studies, the majority of the time was spent on repeat applications. For the National Hand Hygiene Evaluation project, a third of the budget, AUD $348,000 was spent obtaining approval for a study that simply involved surveying nurses about their hand hygiene behaviour. Two of the 50 hospitals identified our research as quality improvement and asked us not to submit a HREC application as it would waste time, whereas another ethical review body required over 7000 pages of documentation.

Our estimates only consider the costs of the research team and not the time of staff at the study sites, ethical review bodies or research governance offices. Nor did we include the financial charges made by some ethical review bodies, for example one charged AUD $660 for the REACH Trial application. We also did not include printing costs or the costs of post-approval monitoring. We also did not include the time spent by the chief investigators writing the protocols or ethical applications. We also only examined overall costs and did not break down the costs into detailed activities as that data was not available.

The estimates for the NHHE were retrospective, and although good records were available on the staff employed and their pay scales, we had to estimate their percent time commitment spent on ethical approvals.

### An established problem

Concern about the time taken for ethical approval is a long-standing issue that has been raised in journals, policies and forums [11–13]. For example, a UK study in 1996 found a similar variability in times to our REACH Trial, as 36 ethical review bodies took between 6 and 208 days [14]. Previous studies have also highlighted the widely varying requirements of ethical review bodies for the same study, and discussed how the burden of ethical processes stymies new research [15, 16]. High costs and long delays have occurred for all types of study designs, including clinical trials [17], observational studies [9] and clinical quality reviews [8].

In 2007, the Australian National Statement on Ethical Conduct in Human Research stated that, “each institution has the further responsibility to adopt a review process that eliminates any unnecessary duplication of ethical review” [2]. Soon after the NHMRC established the Harmonisation of Multi-centre Ethical Review (HoMER) project to try and implement a national approach to multi-centre research. An NHMRC-hosted national forum on clinical trial research governance in 2013 included calls for “streamlining of ethical review processes” and more efficient site-specific approvals, and concluded that better systems should increase Australia’s international competitiveness in clinical trials.

### Recommendations for researchers

Researchers conducting multi-centre studies could prospectively collect the total staff time spent on ethics and site-specific applications. If enough researchers shared this data with our team we could use a meta-analysis to estimate the average cost per study. We could then extrapolate this cost to a national figure that would likely get more political attention than one or two case studies. Given that an estimated 85 % of current research effort is wasted [18], this figure would be part of a larger debate about increasing the value of health and medical research.

Researchers applying for funding for multi-centre trials should ensure their budget includes funding for ethics applications with costs and a timeline that is proportional to the number of centres. Research plans should consider the potential time lag for gaining all approvals and associated impact on trial timings and budgets, especially where commencement of the trial is contingent on all approvals at each site being completed. Researchers acting as grant reviewers need to be accommodating of these long time lines and large budgets.

### Recommendations for improvement

The Australian Government Office for Learning and Teaching commissioned a report into delays in ethical approvals [19], which recommended, “An efficient arrangement for the ethical review of research that has already been ethically reviewed by another institution”. Similarly, a study that considered the variation in decisions of ethical review bodies recommended that after the initial review any subsequent reviews of the same application must be expedited [20]. We strongly support this idea, and this makes sense as the judgement of the original review should be trusted. However, we note that there has been strong support for this idea from researchers and the NHMRC for some time and it has yet to be fully implemented and does not cover site-specific approvals.

The roll-out in 2016 of the new Health Research Authority approval in England shows that reforms to increase efficiency of multi-centre studies are possible [21]. This streamlined process replaced the need for local checks of legal compliance, which is particularly time-saving for multi-centre research. It was piloted to iron out issues, and it publishes regular statistics on the number of applications received and turn-around times.

Relatively simple efficiencies could also be made to the application forms as much of the information on the site-specific form is already in the ethical application.

A key issue is that ethical review bodies are heavily incentivised to reduce risk, rather than save costs for applicants. They are able to externalise costs to those applying for ethical approval in pursuit of some marginal reduction in risks. It is plausible that risk is not reduced at all, but the process of extra review and delay provides an insurance policy against some future unforeseen event. If costs to applicants were somehow made important to ethical review bodies, they might reassess the speed and scope of their work.

### National statistics on review times

We suggest publishing statistics on the time taken for ethical review for every ethical review body and site on a national web page. The statistics would be based on nationally consistent definitions of when the review started and ended. This would allow benchmarking and the identification of review bodies or sites with times that are significantly longer than the typical distribution of times. Our results (Fig. 1) show a large variability, which suggests that national statistics could also show important variability. Results could be stratified by the applications’ risk level (administrative, low and high).

If an ethical review body or site had relatively long review times, then this could be used to identify problems and provide assistance. Advice could be sought from those ethical review bodies and sites with relatively fast times. We acknowledge that people working on ethical review bodies provide their time voluntarily, and they often have to read multiple applications from a wide variety of fields and consider difficult ethical questions that may be poorly communicated or considered by the researchers (leading to delays that are outside the control of the ethical review body). However, one way to reduce their workload is to avoid assessing studies that have already been approved by other bodies.

National statistics on times would allow researchers to better plan how long their applications would take. Some health and medical journals publish similar statistics to fully inform researchers about the expected peer review time.

A national web site of ethical review body times would need financial support and require time from staff at the ethical review bodies. However, given such data are almost wholly electronic, the burden should be minimal, indeed it may already be possible to create these statistics from national online ethics forms.

## Conclusions

This paper adds to the evidence demonstrating the high costs of getting ethical and site-specific approval for multi-centre research. Perhaps we should be resigned to multi-centre applications taking longer than 12 months and build in the costs and times to our research projects. But this means that health improvements are being delayed and funding is being reduced for other research. The public would likely be unhappy at precious research dollars being spent on tasks that delay discovery for no benefit. We think the current systems could be streamlined without any increased risk to public safety.
